# Supplementary material for: Conditional chemoconnectomics (cCCTomics) as a strategy for efficient and conditional targeting of chemical transmission
Source: eLife. 2024 Apr 30;12:RP91927. doi: 10.7554/eLife.91927 (PMC11060718; doi:10.7554/eLife.91927)
Supplement: Figure 3—source data 3. [file elife-91927-fig3-data3.zip › Figure 3 source data3 part 2.zip!/Figure 3-Source data 3-part2/1025-13-CRISPResso_Report_ack040l3.zip!/CRISPResso_on_1025_13/9.Alleles_frequency_table_around_sgRNA_agcacaaacttggcggtgac.pdf]

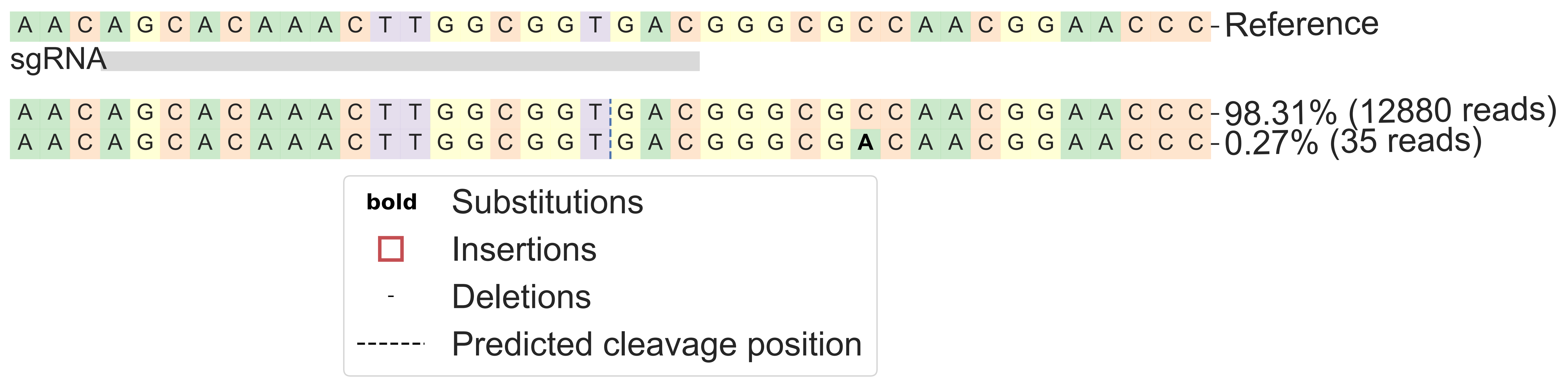
A A C A G C A C A A A C T T G G C G G T G A C G G G C G C C A A C G G A A C C C - Reference
sgRNA
A A C A G C A C A A A C T T G G C G G T G A C G G G C G C C A A C G G A A C C C - 98.31% (12880 reads)
A A C A G C A C A A A C T T G G C G G T G A C G G G C G A C A A C G G A A C C C - 0.27% (35 reads)
bold Substitutions
Insertions
- Deletions
Predicted cleavage position
